# NK Cell Inflammation in the Clinical Outcome of Colorectal Carcinoma

**DOI:** 10.3389/fmed.2015.00033

**Published:** 2015-05-26

**Authors:** Andrea Coppola, Roberto Arriga, Davide Lauro, Maria Ilaria del Principe, Francesco Buccisano, Luca Maurillo, Patrizia Palomba, Adriano Venditti, Giuseppe Sconocchia

**Affiliations:** ^1^Institute of Systems Medicine, University of Rome “Tor Vergata”, Rome, Italy; ^2^Hematology, Department of Biomedicine and Prevention, University of Rome “Tor Vergata”, Rome, Italy; ^3^Laboratory of Tumor Immunology and Immunotherapy, Institute of Translational Pharmacology, CNR, Rome, Italy

**Keywords:** NK cells, colorectal carcinoma, inflammation, CD8^+^ T cells, cooperation, survival

## Abstract

The ability of natural killer (NK) cells to provide protection against myeloid leukemia has been demonstrated in clinical settings. However, whether NK cells play a role in the clinical course of solid tumors is debated. The controversy surrounding the role of NK cells is due, at least in part, to the limited extent of NK cell infiltration found in the tumor bed. Inactivation of NK cells may explain the shortage of NK cells in the microenvironment of colorectal cancer (CRC). Upon NK cell/tumor cell interaction, tumor cells may escape NK cells by creating an immunosuppressive microenvironment, which possibly affects T-cells as well. Such an immunosuppressive microenvironment would hamper the functions of NK and T-cell and reduce NK and T-cell interactions. CRC patients with levels of tumor NK cell infiltration suitable for statistical analysis have been identified. The infiltration of the CRC microenvironment by NK cells, in combination with CD8^+^ T-lymphocytes, has been shown to enhance the prognosis of CRC patients. Here, we discuss the clinicopathological role of NK cells in CRC, and present clinical data indicating a potential supporting role for NK cells in the anti-CRC effects of CD8^+^ T-cells.

## Human Natural Killer Cells

Natural killer (NK) cells are cytotoxic, with proven antitumor activity in mouse models and myeloid leukemia. Recent evidence strongly suggests that the antitumor activity of NK cells is not solely due to their cytotoxic function but also relies on the ability of NK cells to shape a proinflammatory microenvironment. NK cells are defined by three main characteristics: (i) a lack of a recombinant-activating gene (RAG)-dependent rearranged antigen receptor, (ii) lymphoid morphology, and (iii) expression of myeloid cell surface markers. NK cells belong to group I members of innate lymphoid cells. They are well-known producers of interferon gamma (IFN-γ) and proinflammatory cytokines, but they are unable to synthesize T_H_17 and T_H_2 cell-associated cytokines. Based on the intensity of expression of the neural cell adhesion molecule 1 (NCAM1), known as CD56 molecule, on the cell surface, two subsets of NK cells have been identified. The first subset, referred to as CD56^dim^ NK cells, is characterized by low levels of CD56 expression in about 90% of Fcγ receptor IIIA (CD16)^high^ NK cells. The second subset, referred to as CD56^bright^ NK cells, is characterized by high levels of CD56 expression in about 10% of CD16-negative NK cells (1). The latter population mainly produces regulatory cytokines, and the former mediates natural and antibody-dependent cellular cytotoxicity (ADCC). CD56 is a broad marker of NK cells; however, it is also expressed by NK T-cells, (2) dendritic cells, and a small subset of human monocytes (3). Thus, the CD56 antigen is not an exclusive marker for phenotypic and functional characterization of human NK cells.

Natural killer cells originate in bone marrow from CD34^+^Lin-CD45RA^+^CD10^+^ common lymphoid progenitor cells. In the absence of bone marrow stroma, the differentiation of CD34^+^ cells into NK cells requires interleukin-2 (IL-2) or IL-15 and stem cell factors. IL-15 appears to be the most relevant cytokine for NK cell differentiation, and is synthesized by bone marrow stromal cells (4). In the early differentiation stage, NK cells have a CD56^bright^ CD94/NKG2A^+^CD16^−^granzyme A+ phenotype. Early differentiated NK cells have a defective cytotoxic function but can exert antiproliferative activity against tumor cells (5).

Natural killer-cell-mediated cytotoxicity is regulated by a balance between signals generated by natural and accessory triggering receptors and inhibitory receptors. NK cells use an array of cytotoxic receptors capable of recognizing cell surface ligand(s) expressed on tumor and normal cells. Some of these receptors, including NKp30, NKp44, and NKp46 (6), are preferentially expressed on NK cells, and are involved in the recognition of leukemia and solid tumor cells. CD16 is also a primary NK activating receptor that mediates both natural cytotoxicity by unknown ligand(s) and ADCC (7). The role of the CD16 antigen in tumor immunotherapy has been the focus of recent attention in the field of tumor immunotherapy, as clinically relevant therapeutic monoclonal antibodies (mAbs) exert their antitumor function through an ADCC-mediated mechanism. NKG2D is another critical receptor mediating NK cell killing of solid tumor cells. It binds to the major histocompatibility complex class I polypeptide-related sequence A/B (MICA/B) and to the UL16 binding protein expressed on solid tumor cells and leukemic cells, respectively. DNAX accessory molecule-1 (DNAM-1) is an additional activating receptor that binds to Nectin-2 and the poliovirus receptor, CD155/PVR. Nectin-2 is expressed in cell junctions of non-malignant cells, and CD155/PVR is expressed by some myeloid and CD4^+^ T-cells. Nectin-2 and CD155/PVR have been identified in a variety of solid tumor cells. NKp80 is the ligand of the activation-induced C-type lectin, and 2B4, which recognizes the CD48 antigen, are involved in the recognition of leukocytes and endothelial, leukemia, and solid tumor cells (8). Additional cell surface molecules, such as CD2, CD69, CD44 (9), and CD38 (10), are involved in NK cell activation. These molecules have been identified with conventional mAbs, which redirect NK cell cytotoxicity against Fc receptor-positive cells. Bispecific antibodies have been utilized for redirecting NK cells against Fc receptor-negative cells.

Natural killer cells use polymorphic killer inhibitory receptors (KIRs) to prevent autoimmunity. Under these circumstances, full engagement of KIRs with human leukocyte antigen (HLA) class I leads to the inhibition of NK-cell-mediated cytotoxicity. Human KIRs are separated into two groups: immunoglobulin-like receptors and heterodimers, characterized by an invariant protein termed CD94 and a variable C-type lectin subunit, also known as NKG2 (CD94/NKG2). KIRs have intracellular tyrosine inhibitory motifs in their cytoplasmic domains. To date, investigators have identified 14 KIRs. Early in 2000, a dedicated committee established a useful and versatile nomenclature system for KIRs. The digit following the acronym (KIR) represents the number of the Ig-like domains in the molecule. The letter “D” stands for the domain, and the letter “L” and “S” stand for long and short cytoplasmic tails, respectively. The last digit indicates the gene that codes for the protein. Inhibitory KIRs possessing two or three Ig-domains are referred to as KIR2DL and KIR3DL, respectively. The former has specificity for HLA-C, and the latter has specificity for HLA-A/B. Due to polymorphisms at positions 77 and 80 in the α1-domain of the β chain, KIR2DL1 and KIR2DL2/KIR2DL3 recognize different groups of HLA-class I alleles. KIR2DL1 binds to HLA class I alleles, defined as C2 (Cw2, Cw4, Cw5, and Cw6). KIR2DL2 and KIR2DL3 bind to HLA-class I alleles, designated as C1 (Cw1, Cw3, Cw7, Cw8). KIR3DL2 binds to HLA-A3 and HLA-A1, and KIR3DL1 interacts with the HLA-Bw4 determinant. Upon specific binding with one or a group of HLA class I molecules, these receptors recruit tyrosine phosphatases, leading to inhibition of cellular functions (11). “L” KIRs are flanked by short “S” KIRs. “S” KIRs are “L” KIR homologous in the extracellular region, but they possess a short intracellular tail that lacks intracellular tyrosine inhibitory motifs, and are associated with a protein tyrosine-kinase-binding protein also known as DAP12. These molecules can function as activating receptors.

## NK and T-Cell Cooperation in the Clinical Development of Colorectal Cancer

With the exception of cancers developing in colonic tissues affected by inflammatory bowel diseases, there is a consensus that inflammation may play a role in the clinical course of colorectal cancer (CRC). Infiltration of the CRC tumor microenvironment by tumor-associated macrophages (12–14), CD8^+^ T-cells (15), and CD4^+^ regulatory T-cells (16) is an independent favorable prognostic factor for CRC. Among solid tumors, the protective role of tumor-associated macrophages in CRC is unique and likely reflects an altered balance between M1 and M2 macrophages. M2 macrophages promote tumor progression by enhancing angiogenesis and inhibiting an immune response against tumors, whereas M1 macrophages promote a proinflammatory microenvironment by releasing IL-1β, IL-6, IL-12, and tumor necrosis factor alpha (TNF-α). Indeed, a recent study demonstrated that the presence of high M1 macrophage infiltration in the CRC tumor microenvironment was associated with improved overall survival (OS) in a stage-dependent manner (17).

To date, there is conflicting information about the clinical relevance of NK cell infiltration in CRC. Although a number of tumor cells of different histological origin express high levels of the NKG2D ligand complex MICA/B, NK cells in the solid tumor microenvironment are barely detectable. A paucity of NK cells in the solid tumor milieu represents the main hurdle to determine whether NK cells play a role in the clinical course of solid tumors (18). Recently, we and other investigators have focused on the investigation of the clinical impact of NK cell infiltration on the prognosis of CRC. An early study involving 157 patients showed that patients with tumor-node-metastasis stage III disease with extensive CD57^+^ NK cell infiltration survived longer than those with low infiltration (19). In a study of 93 patients, Menon et al. showed that patients with elevated levels of CD56^+^ or CD57^+^ NK cell infiltration had longer disease-free survival (DFS) than those with lower levels of infiltration. Marechal et al. examined the CD56^+^ cell content of 68 primary tumors of CRC patients with tumor-node-metastasis stage IV. In their study, the patients were treated with chemotherapy, with or without cetuximab, a therapeutic chimeric mAb. They concluded that the presence of CD56^+^ cells in the tumor milieu was an independent favorable prognostic factor, and that it was associated with prolonged progression-free survival only in the group of cetuximab-treated patients. An additional study by Gulubova et al. of 34 CRC patients showed that NK cell infiltration was decreased in livers harboring metastases as compared to that of normal organs. However, no information about the DFS survival and OS of the CRC patients was provided. Finally, a phenotypic analysis of NK cell-activating receptors of peripheral blood NK cells in CRC patients indicated that these molecules were significantly down-regulated as compared to those of healthy controls. Moreover, a reduced percentage of NKG2D and perforin-positive NK cells were associated with a poor histological grade (20). Thus, NK cell infiltration has been associated with a favorable prognosis in only two studies. Conversely, Sandel et al. and Halama et al. recruited 88 and 112 CRC patients, respectively, and both showed that NK cell infiltration had no effect on the clinical development of the disease (21). Therefore, the clinical relevance of NK cell infiltration in the prognosis of CRC remains elusive.

The sample size utilized in the aforementioned studies was clearly very small. Thus, we investigated NK cell infiltration in CRC by staining a tissue microarray of 1414 CRC punch biopsies, using CD56 or CD57 as NK cell markers. Immunohistochemistry analysis indicated that 38 and 15% of punch biopsies showed evidence of CD56^+^ or CD57^+^ cell infiltration, respectively. We found that NK cell infiltration had no prognostic effect on the clinical course of the patients. Another study found that NK cell infiltration was not associated with mismatch repair-deficient or mismatch repair-proficient CRC tumor status (14). Recent studies have shown that NK cells might collaborate with CD8^+^ T-cells, and that such crosstalk may initiate or intensify an antigen-driven T-cell response. These findings prompted us to investigate whether CRC infiltration by NK cells, in combination with T-cells, improved the prognosis of CRC patients. NK and T-cells were stained with biotinylated mAb specific for CD56 or T-cell markers, such as CD3, CD4, and CD8, and these markers were detected using immunoperoxidase methodology. Receiver operating characteristic curve analysis identified cut-off scores for negative (≤4 NK cells) and positive (>4 NK cells) CRC. NK cells were identified in 423 of 1410 CRC tumor biopsies, but only132 (9%) contained >4 NK cells. This study demonstrated that the OS of CRC patients with CD56^−^/CD8^+^ cell infiltration was significantly higher than that of patients with CD56^−^/CD8^−^ and CD56^+^/CD8^−^ cell infiltration. Most importantly, patients bearing tumors with CD56^+^ and CD8^+^ cell infiltration had the best OS. NK cell infiltration, combined with CD3^+^ and CD4^+^ T cell and macrophage infiltration, did not appear to affect the clinical development of the disease. In addition, two lines of evidence suggested that CD56^+^ cells detected in CRC microenvironment belonged to an NK cell population. First, there was no correlation between CD8^+^ T-cells and CD56^+^ cell infiltration. Second, flow cytometry analysis of enzymatically dissociated CRC tumor cells revealed a robust association between CD56^+^ cells and cells expressing more specific NK cell markers, such as NKp46. These results indicate that the presence of both NK cells and CD8 cells in the CRC microenvironment has a favorable prognostic impact in CRC (22).

## NK Cells that Escape CRC Cell-Induced Inactivation may Shape a Proinflammatory Tumor Microenvironment

The results of the aforementioned clinical studies raise the question whether the mechanisms of immune evasion of CRC cells limit the antitumor immune response. *In vivo* and *in vitro* studies have indicated that solid tumors induce both NK cell and T-cell dysfunction by producing immunosuppressive molecules. Published reports have shown that indoleamine 2,3-dioxygenase and prostaglandin E2 produced by melanoma cells modify the NK cell phenotype and impair the functions of NK cells (23). Solid tumor cells escape NK cell recognition by utilizing transforming growth factor beta-1 (24) and lymphocyte function-associated antigen 1 (25). This may explain the minimal NK cell infiltration in the solid tumor microenvironment (14, 18, 25). Importantly, the immunosuppressive effects of tumor cells may affect NK cells and T-lymphocytes, as well as their cooperation.

To date, there is insufficient information about the contribution of intratumoral NK cells to CRC outgrowth and progression. However, a combination of immunohistochemistry analysis and *in vitro* studies, where NK cells were cultured with CRC and T-cells and monocytes, has provided valuable information on the mechanisms underlying CRC cell-mediated NK cell dysfunction. Coculture of NK cells with CRC cells (HCT116, LS-180, COLO-205, and SW-480) induced NK cell apoptosis and CD16 downregulation (26). Following NK cell interaction with CRC cells, CD16 was strongly downregulated, which was likely mediated by metalloprotease (MMP) activation, leading to CD16 cell surface trimming and shedding in the culture supernatant. Interestingly, the use of MMP inhibitors reduced CD16 antigen downregulation and helped to prevent host immunosuppression by improving NK cell-mediated ADCC and CD16-mediated killing of tumor cells (27).

**Figure 1:**
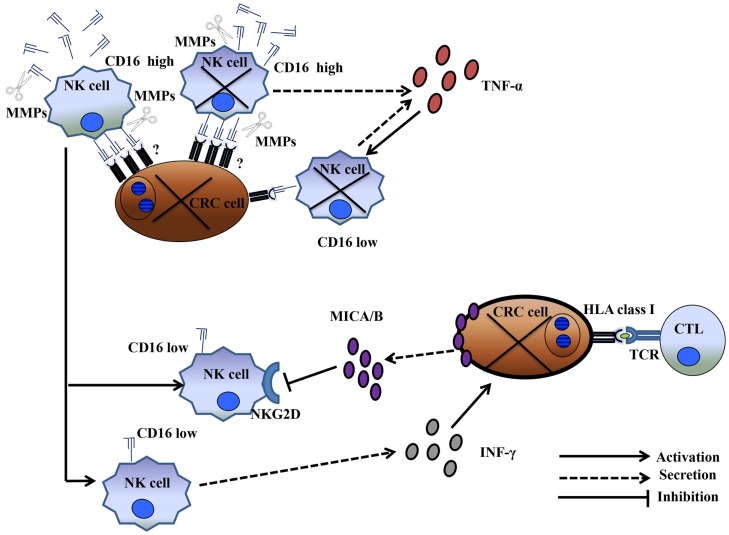
**CD16^low^ NK cells surviving CRC-cell-induced dysfunction boosted the CD8^+^ T-cell-dependent anti-CRC tumor immune response**. CD16^high^ NK cell conjugation with CRC cells led to the elimination of both NK and CRC cells. MMP activation and TNF-α production resulted in a population of CD16^low^ NK cells that survived cancer-cell-contact and remained functionally active. These cells produced IFN-γ, leading to upregulation of HLA class I antigen(s) on cancer cells. HLA class I upregulation enhanced tumor antigen presentation to CD8^+^ T-cells. Conversely, the cancer cells protected themselves from CD16^low^ NK-cell-mediated cytotoxicity through the release of soluble MICA/B.

**Figure 2:**
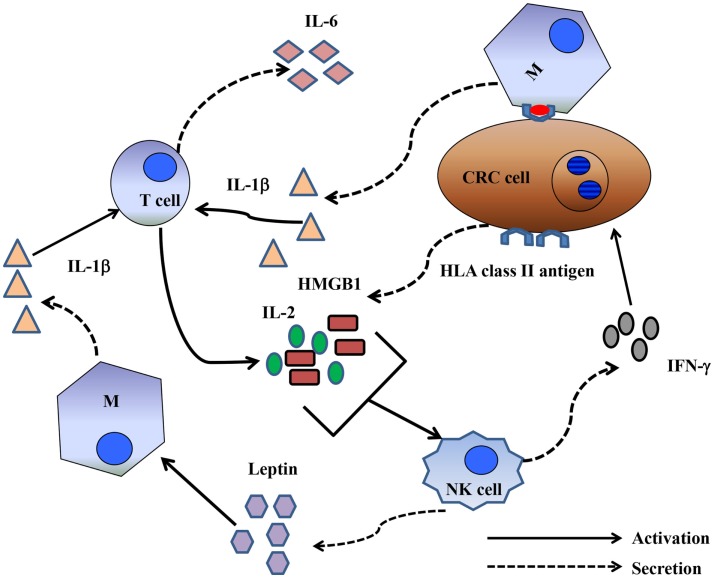
**Crosstalk between NK cells and T-cells could shape a proinflammatory immune response**. Activated T-cells produced IL-2, and CRC cells produced HMGB1. IL-2 and HMGB1 in combination activated NK cells to produce leptin and IFNγ. The latter induced *de novo* expression of HLA class II antigens by CRC cells. HLA class II antigens, presumably by interacting with the CD4 antigen, may promote IL-6 production by macrophages. Leptin may then directly stimulate macrophages to produce IL-1β, which then stimulates T-cells to synthesize IL-6.

The mechanism by which CRC cells induce NK cell depletion and apoptosis is poorly understood. However, the CD16 antigen may play a significant role in the induction of NK cell apoptosis. In a previous study, triggering the CD16 antigen by anti-CD16 mAbs induced a CD16^−^CD56^+^CD69^+^Fas^+^ NK cell phenotype, as well as TNF-α production, leading to NK cell apoptosis. Interestingly, TNF-α induced cell death failed to affect a minor population of NK cells, which, in the presence of IL-2, remained cytotoxic. In addition, NK cell apoptosis was abrogated *in vitro* by the use of an anti-TNF-α mAb (28). Although this is an intriguing observation, the production of TNF-α may not be exclusively involved in the mechanism of CRC-cell-induced NK cell apoptosis because a general caspase inhibitor failed to inhibit HCT-116-cell-induced NK cell apoptosis. In contrast, similar to the results described by Jewett et al., another study reported that an NK cell population that escaped colon cancer inactivation was CD16^low/negative^ (25). An important question relating to the role of a proinflammatory microenvironment in improving the clinical course of CRC is whether CD16^−^/CD56^+^ NK cells are involved in bidirectional crosstalk with CRC cells and CD8^+^ T-cells. Emerging data suggest that NK cells interact with cancer cells and cellular components of the immune system, including dendritic cells, macrophages, and T-cells. Available clinical data strongly suggest that NK cells, unlike T-cells, may not represent a major anticancer cell population. Alternatively, NK cells could have an immunoregulatory function, possibly by shaping a proinflammatory CRC microenvironment supporting CD8^+^ T-cells. Two mechanisms may account for the immunoregulatory properties of NK cells. The first involves CD16^low/negative^ CD56^+^ NK cells that survive CRC-cell-induced apoptosis. These cells can synthesize and release IFN-γ in the CRC microenvironment. IFN-γ can induce upregulation of HLA class I antigens in CRC cells, enhancing CRC antigen presentation to CD8^+^ cytotoxic T-lymphocytes, which, in turn, may eliminate CRC cells (Figure 1). The second suggests crosstalk between activated T-cells and CRC cells. Activated T-cells produce IL-2. IL-2 and the high mobility group box (HMGB)1 (29) released by CRC cells stimulate NK cells to produce IFNγ and leptin (30). IFNγ induces HLA class II antigen expression in CRC cells. The interaction of HLA-class II antigens with macrophages, in the presence of leptin, triggers macrophages to secrete IL-1β (31). IL-1β induces IL-6 synthesis and release by T-lymphocytes, contributing to proinflammatory polarization of the tumor microenvironment a (32) (Figure 2).

## Concluding Remarks

Although the clinical role of NK cells remains controversial, the observation that NK cells seems to support the anti-CRC activity of CD8 T-lymphocytes highlights the need for more studies in this area. To this end, investigators must define advanced experimental strategies aimed at (i) unveiling details about the phenotype and function of intratumoral NK cells, (ii) investigating how NK cells crosstalk with T-cells, and (iii) identifying ways to inhibit CRC cell-induced NK cell dysfunction. The accomplishment of these tasks will be critical for the development of successful NK cell-based immunotherapy for CRC.

## Author Contributions

AC and RA equally contributed to this work. AC, RA, and GS conceived and wrote the manuscript. DL, MP, FB, PP, AV, LM, and GS critically revised and improved the manuscript writing. All authors fully agree with the manuscript’s content.

## Conflict of Interest Statement

The authors declare that the research was conducted in the absence of any commercial or financial relationships that could be construed as a potential conflict of interest.
